# Genetic diversity of the *Pvk12* gene in *Plasmodium vivax* from the China-Myanmar border area

**DOI:** 10.1186/s12936-016-1592-z

**Published:** 2016-11-04

**Authors:** Shuang Deng, Yonghua Ruan, Yao Bai, Yue Hu, Zeshuai Deng, Yongshu He, Rui Ruan, Yanrui Wu, Zhaoqing Yang, Liwang Cui

**Affiliations:** 1 Department of Pathogen Biology and Immunology, Kunming Medical University, Kunming, 650500 Yunnan Province China; 2 Department of Pathology, Kunming Medical University, Kunming, 650500 Yunnan Province China; 3 Department of Pharmacology, Kunming Medical University, Kunming, 650500 Yunnan Province China; 4 Department of Cell Biology and Medical Genetics, Kunming Medical University, Kunming, 650500 Yunnan Province China; 5 Department of Orthopedics, The First Affiliated Hospital, Kunming Medical University, Kunming, 650500 Yunnan Province China

**Keywords:** Malaria, *Plasmodium vivax*, *Pvk12* gene, Polymorphism, Molecular surveillance

## Abstract

**Background:**

*Plasmodium falciparum* resistance to artemisinin emerged in the Greater Mekong Sub-region has been associated with mutations in the propeller domain of the kelch gene *Pfk13*.

**Methods:**

Here the polymorphisms in *Pvk12* gene, the orthologue of *Pfk13* in *Plasmodium vivax,* were determined by PCR and sequencing in 262 clinical isolates collected in recent years (2012–2015) from the China-Myanmar border area.

**Results:**

Sequencing of full-length *Pvk12* genes from these isolates identified three synonymous mutations (N172N, S360S, S697S) and one non-synonymous mutation M124I, all of which were at very low prevalence (2.0–3.1%). Moreover, these mutations were non-overlapping between the two study sites on both sides of the border. Molecular evolutionary analysis detected signature of purifying selection on *Pvk12*.

**Conclusions:**

There is no direct evidence that *Pvk12* is involved in artemisinin resistance in *P. vivax,* but it remains a potential candidate requiring further investigation. Continuous monitoring of potential drug resistance in this parasite is needed in order to facilitate the regional malaria elimination campaign.

**Electronic supplementary material:**

The online version of this article (doi:10.1186/s12936-016-1592-z) contains supplementary material, which is available to authorized users.

## Background

Although global malaria incidence and death rates have decreased in recent years, malaria remains one of the biggest threats to populations living within the tropical and sub-tropical world. In the Greater Mekong Sub-region (GMS) of Southeast Asia, a regional malaria elimination plan has been endorsed, aiming to eliminate *Plasmodium falciparum* malaria by 2025 and all malaria by 2030 [1]. This ambitious goal is met with challenges, especially the recently developed artemisinin resistance among *P. falciparum* populations in this region [2–4]. Further complication of the situation results from the increasing prevalence of *Plasmodium vivax* in this region, a parasite that is resilient to current control measures [5]. Although chloroquine (CQ) remains the primary treatment option for *P. vivax* infections, the emergence of CQ resistance has led to the use of artemisinin-based combination therapy (ACT) for the treatment of vivax malaria in Indonesia [6]. Monitoring drug resistance in *P. vivax* populations is important for eliminating all malaria from the GMS.

In regions co-endemic for *P. falciparum* and *P. vivax*, both species can share the same vectors and human hosts, and are often subject to similar forces of natural selection [7, 8]. Several studies showed that anti-malarial drug pressure induces strong selection on both parasite species [9, 10]. It is thus assumed that the widespread use of ACT for treating *P. falciparum* infection may exert similar collateral selective pressure on *P. vivax* populations. Recently, mutations in the propeller domain of the *Pfk13* gene have been incriminated as important determinants of artemisinin resistance in *P. falciparum* [11–13]. Although ACT is still highly efficacious for treating vivax malaria [14, 15], vigilance is required on potential emergence of resistance in this parasite [16]. A recent study aiming to determine whether similar mutations mediating artemisinin resistance are also present in the *Pfk13* orthologue in *P. vivax* (here named *Pvk12* gene as this gene is located on chromosome 12) found that parasites with a *Pvk12* mutation (V552I) was circulating in Cambodia at a very low frequency [17]. To further examine the genetic diversity of this gene in the GMS, recent collections of clinical isolates of *P. vivax* from both sides of the China-Myanmar border were examined. This region has a very long history of artemisinin deployment and there is an indication that CQ efficacy for treating *P. vivax* malaria is decreasing [18]. Here *Pvk12* genes in 262 *P. vivax* parasite isolates were sequenced and molecular evolution analyses were performed to determine whether this gene is subject to potential selection.

## Methods

### Collection of parasite clinical isolates

*Plasmodium vivax* samples were collected from malaria patients with acute *P. vivax* infections attending malaria clinics located on both sides of the China-Myanmar border. *Plasmodium vivax* infection was diagnosed by microscopy of Giemsa-stained thick and thin blood films. A total of 162 samples were obtained from 13 clinics around Laiza township, Kachin State, Myanmar during the high transmission season (June to September) in 2015. One-hundred samples were collected in 2012–2013 from Tengchong County, Yunnan Province, China. The standard treatment for *P. vivax* malaria is CQ (25 mg/kg, divided into three days) and primaquine (0.25 mg/kg/day for 14 days in Kachin State; 0.375 mg/kg/day for 8 days in Yunnan Province) [19]. On both sides of the border, malaria transmission has been reduced in recent years [20, 21]. Especially, *P. falciparum* malaria incidence has experienced a sharp decline [22, 23], possibly due to the extensive use of ACT. To collect finger-prick blood on filter papers, written informed consent was obtained from the participants or their guardians. This study was approved by the Institutional Review Board of Kunming Medical University.

### DNA extraction, gene amplification and sequencing analysis

Genomic DNA was extracted using the High Pure PCR Template Preparation Kit (Roche, Germany) and eluted in 50 µl of H_2_O. Parasite samples were genotyped by PCR/RFLP of two polymorphic genes *msp3α* and *msp3β* to distinguish single from mixed-strain infections [24, 25]. Only infections containing single *P. vivax* strains by genotyping were used for sequencing analysis. The full-length *Pvk12* gene was amplified by nested PCR using 2X TSINGKE™ Master Mix (Beijing Tsingke Biotech, China) containing high-fidelity Pfu DNA polymerase with outer primers K13P1F (5′-CCATACTGGCTGCACCTGCTT-3′) and K13P1R (5′-GTAGTGGCAGTGGAGGAGAG-3′), and nested primers K13P2F (5′-CCACGGAACAGATGAATCTTC-3′) and K13P2R (5′-AAACCCGAGAAAGTTGTAGCA-3′). PCR reactions were performed in 25 with 1 µl DNA template, 0.5 μM of each primer, 12.5 μl Master Mix, and 10.5 μl H_2_O. For the primary reaction the following cycling parameters were used: 5 min at 94 °C, 35 cycles at 94 °C for 30 s, 63 °C for 30 s, 72 °C for 90 s, and final extension for 7 min at 72 °C. For nested PCR, 1 μl primary PCR product was used as template and the following cycling parameters were used: 5 min at 94 °C, 35 cycles at 94 °C for 30 s, 57 °C for 30 s, 72 °C for 90 s, and final extension for 7 min at 72 °C. Sequencing was performed on both strands and sequences were assembled by using DNASTAR (WI, USA) with manual editing. Alignment of DNA sequences were performed using MEGA 6.0 [26] and BioEdit (version 7.2.5) with the *Pvk12* sequence of Salvador I (Sal-I) genome (PVX_083080) as the reference. The five haplotypes of *Pvk12* gene derived from this study have been submitted to GenBank under the accession numbers (KX961684–KX961688).

### Molecular evolutionary analysis of *Pvk12*

A total of 333 full-length *Pvk12* sequences were analysed, of which 262 samples were from the current study. The remaining 71 sequences including four from Oceania, 11 from North America, one from Africa, 34 from South America, and 21 from Southeast Asia were downloaded from PlasmoDB [27], as a result from the *P. vivax* hybrid selection sequencing project [28]. The sequences were aligned with the Sal-I reference sequence using ClustalW implemented in MEGA 6.0. SNP information was used to generate haplotypes and to calculate haplotype diversity. To determine the genetic diversity of *Pvk12* gene, the average pairwise nucleotide diversity (π) and the average number of segregating sites (θ) were calculated. To detect signatures of natural selection, the number of non-synonymous substitutions per non-synonymous site (*d*N) and synonymous substitutions per synonymous site (*d*S) were estimated. A codon-based Z test was performed to calculate possible departure from neutrality with the variance of difference between *d*N and *d*S being computed by the bootstrap method with 500 replicates [29]. This neutrality test was further corroborated using Tajima’s D and Fu’s Fs tests. All molecular evolution tests were performed using MEGA 6.0. Pairwise linkage disequilibrium (LD) was also used to determine the degree of random association between different mutations within this gene. The correlation coefficient (*R* ^*2*^) between paired alleles was estimated using DNASP v5.0 and Arlequin 3.5, and the significance of each association was determined using the χ^2^ test after Bonferroni correction.

## Results

### Genetic diversity of *Pvk12* from the two geographical regions

**Fig. 1:**
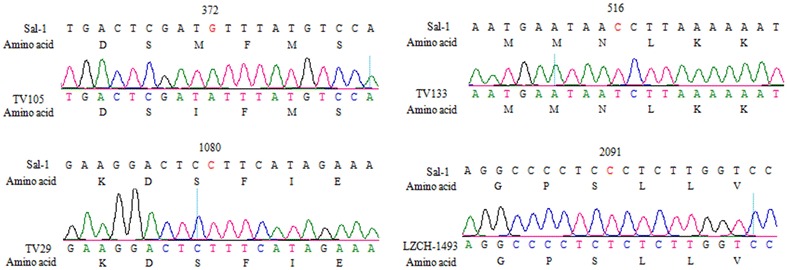
Example chromatograms of the *Pvk12* gene showing synonymous mutations at nucleotide positions 516, 1080 and 2091, and non-synonymous mutations at position 372. Included are the *Pvk12* sequences in the Sal-I strain and parasite isolates TV105, TV133, TV29 and LZCH-1493

Two hundred and sixty two full-length *Pvk12* sequences (2139 bp) in parasite samples from two regions along the China-Myanmar border were obtained. Compared with the Sal-I reference sequence, one synonymous SNP at nucleotide 2091 was observed in five (3.1%) isolates from the Kachin samples, while two different synonymous SNPs at nucleotide 516 and 1080, respectively, were identified in four (2%) isolates from the Tengchong samples (Table 1; Fig. 1). In addition, one non-synonymous mutation, M124I, was found in two isolates from Tengchong, whereas no non-synonymous mutation was identified in the Kachin samples. Altogether, the frequency of parasites containing *Pvk12* mutations was very low, making up of only 4.2%. In comparison, 71 downloaded *Pvk12* sequences were all the same as the wild type reference sequence. Consistent with this result, genetic diversity of *Pvk12* gene represented by the π and θ values was extremely low for the China-Myanmar border samples (Table 2). Similarly, only five haplotypes were observed with the wild type as the predominant. Among the parasite isolates from Tengchong, four haplotypes were observed, giving a haplotype diversity of 0.116. The haplotypes of these two regions were completely different without overlap. Except for the wild type, other haplotypes containing single mutations all were very rare. Furthermore, pairwise LD analysis revealed no significant associations between different alleles (see Additional files 1, 2; *P* > 0.05, χ^2^ test).

**Table 1:** Prevalence of point mutations in the *Pvk12* gene of *Plasmodium vivax* isolates from the two regions along the China-Myanmar border

Point mutations		Prevalence [N (%)]	
Nucleotide	Amino acid	Kachin (N = 162)	Tengchong (N = 100)
G372A	M124I	–	2 (2.0%)
C516T	N172N	–	2 (2.0%)
C1080T	S360S	–	2 (2.0%)
C2091T	S697S	5 (3.1%)	–

**Table 2:** Genetic diversity and summary statistics of *Pvk12* gene

Regions	No. isolates	H	θ	π	dN–dS	Tajima’s D	Fu’s Fs
Kachin	162	2	0.000083	0.000028	−1.071	−0.69076	−0.91123
Tengchong	100	4	0.000271	0.000056	−1.275	−1.42652*	−3.88033*
Total	262	5	0.000304	0.000039	−1.579	−1.49534*	−6.52209*

*H* number of haplotypes

* *P* < 0.05

### Signature of purifying selection

Several molecular evolutionary analyses were performed to assess the role of natural selection on *Pvk12*. When all samples were combined, both Tajima’s D and Fu and Li’s Fs were significantly deviated from neutrality (Table 2) and suggested of purifying selection. The dN–dS analysis also showed higher prevalence of synonymous than non-synonymous mutations, albeit the value was not statistically significant. Interestingly, when samples from the two regions were analysed separately, purifying selection on this gene was only evident in isolates from Tengchong, China (Table 2). Although the Tajima’s D and Fu and Li’s F values for the Kachin samples did not reach significance, they had the same trend as for the Tengchong samples.

## Discussion

Mutations in the propeller domain of *Pfk13* gene have been associated with artemisinin resistance in *P. falciparum*, which is manifested as delayed parasite clearance after artemisinin treatment [11, 12]. A recent analysis of worldwide *P. falciparum* populations identified numerous non-synonymous mutations with marked geographic disparity in their frequency and distribution, which may reflect the demographic history of the parasite populations and different drug use histories [30]. In sharp contrast, studies conducted to date in the GMS, where artemisinin resistance has emerged in *P. falciparum,* showed a highly conserved *Pvk12* gene [17, 31]. In Cambodia where *Pfk13* mutations were highly prevalent, analysis of 284 *P. vivax* isolates identified only two isolates with a non-synonymous mutation, V552I [17], although these parasites may have been exposed to higher drug selection pressure. Similarly, analysis of 66 *P. vivax* samples from central China identified a G581R mutation in one sample (Wang et al. unpublished). In comparison, this analysis of 262 full-length *Pvk12* sequences only identified that two parasite isolates (2%) collected from Yunnan’s Tenchong County contained a non-synonymous mutation, M124I, which is located near the N-terminal region. Thus, mutations identified here, being outside of the propeller domain, are unlikely involved in artemisinin resistance even if *Pvk12* is presumed to play an analogous role to *Pfk13* in resistance. Furthermore, although the recent genomic studies of world *P. vivax* populations identified five non-synonymous mutations in *Pvk12* (N25I, S253T, V552I, V652L, and A710E) in 228 samples, all of these mutations were rare mutations [31]. Specifically, the N25I mutation was identified only in 2% of the western Thai samples (N = 89), whereas the S253T mutation was found only in 8% of the Papua Indonesia samples (N = 55), and each of the three other mutations was present only in one sample. Though some of these mutations are located in the propeller domain, their functions still need to be evaluated individually as mutations within the propeller domains such as A578S in *Pfk13* may not necessarily be associated with artemisinin resistance [12].

The observed deficiency of mutations in the *Pvk12* gene is in sharp contrast to *Pfk13* from the sympatric *P. falciparum* populations [11, 30]. While it is unknown whether *Pvk12* and *Pfk13* are functionally equivalent, the high level of conservation of *Pvk12* suggests that (1) *Pvk12* may play a different role in parasite biology even if *Pvk12* has a similar function to *Pfk13* and its conservation is maintained by functional constraints (purifying selection) and, (2) if *Pvk12* is involved in artemisinin resistance, the different mutation rates in *Pfk13* and *Pvk12* may suggest different drug selection pressures on these two parasites. Likewise, there is evidence that *pfk13* is under natural selection as non-synonymous mutations exceed synonymous mutations [32]. In contrast, *Pvk12* appears to be under purifying selection, though the limited polymorphisms at four positions of *Pvk12* in 11 of 262 samples analyzed here preclude a robust evolutionary analysis. To date the functions of *Plasmodium* *k13* orthologues are still unknown, but their similarity in sequence to the human KEAP1 protein, which is involved in the regulation of the antioxidant responses [33], suggest that they might have similar functions. In *P. falciparum*, it was found that the *Pfk13* is linked to the phosphotidylinositol-3-phosphate kinase (*PI3K*) pathway and artemisinin-conferring *Pfk13* mutations are associated with increased *PI3K* activity and higher levels of the product phosphatidylinositol-3-phosphate [34]. If a similar pathway linking *k13* and *PI3K* works in both parasite species, drug selections may be imposed on different enzymes within the same pathway. Interestingly, divergent selections have been identified on the phosphoinositide-dependent kinase PDK-1 among *P. vivax* populations, an enzyme participating in the *PI3K* pathway [28]. It is unknown whether this observation suggests that artemisinin family drugs might have acted on the *PvPDK*-*1* gene instead of *Pvk12* gene in *P. vivax*. It is also possible that the highly conserved *Pvk12* gene among worldwide *P. vivax* populations may suggest that *Pvk12* may have nothing to do with artemisinin resistance, like that the *pvcrt*-o gene is not a major player in CQ resistance in *P. vivax* as compared to the critical role of its orthologue *pfcrt* in mediating CQ resistance in *P. falciparum*.

Mutations within *Pvk12*, though rare, displayed quite significant geographical disparities. Three different non-synonymous mutations have been identified in parasite populations from different parts of the GMS [17, 31]. Considering evidence of independent emergence of artemisinin resistance-conferring *Pfk13* mutations in different areas of the GMS [35], this difference in *Pvk12* may indicate different demographic histories and the presence of gene flow barriers among these parasite populations. It could also reflect different drug use histories for *P. falciparum* malaria, which may have exerted collateral selection on *P. vivax*, given that the front-line treatment for *P. vivax* malaria in most GMS countries remains as CQ and primaquine. Nevertheless, the collateral selection by artemisinins in the case of mixed *P. falciparum*/*P. vivax* infections that are treated by ACT should be minimal. *Plasmodium vivax* parasites reappearing after ACT treatment may be the relapsing parasites resulted from the awakening hypnozoites, and thus further transmission probably will be from these unselected parasites. In this regard, future surveillance on artemisinin resistance in *P. vivax* may need to focus on areas such as Indonesia where ACT has replaced CQ for treatment of vivax malaria, though recent analysis of a limited number of samples did not detect mutations in the propeller domain of *Pvk12* in the Papua Indonesia samples [31].

## Conclusions

*Pvk12* only displayed very limited genetic diversity in parasite populations from the China-Myanmar border area, as well as from other regions of the GMS, where artemisinin resistance is *P. falciparum* has emerged recently. *Pvk12* is not implicated in drug resistance nor is it recommended for molecular surveillance for drug resistance.

## Additional files

**Additional file 1.** Linkage disequilibrium analysis of total samples.

**Additional file 2.** Linkage disequilibrium analysis of the Tengchong samples.

## References

[1] WHO (2015). Strategy for malaria elimination in the Greater Mekong Subregion (2015–2030).

[2] Noedl H, Se Y, Schaecher K, Smith BL, Socheat D, Fukuda MM (2008). Evidence of artemisinin-resistant malaria in western Cambodia. N Engl J Med.

[3] Dondorp AM, Nosten F, Yi P, Das D, Phyo AP, Tarning J (2009). Artemisinin resistance in *Plasmodium falciparum* malaria. N Engl J Med.

[4] Phyo AP, Nkhoma S, Stepniewska K, Ashley EA, Nair S, McGready R (2012). Emergence of artemisinin-resistant malaria on the western border of Thailand: a longitudinal study. Lancet.

[5] Sattabongkot J, Tsuboi T, Zollner GE, Sirichaisinthop J, Cui L (2004). *Plasmodium vivax* transmission: chances for control?. Trends Parasitol.

[6] Baird JK (2011). Resistance to chloroquine unhinges vivax malaria therapeutics. Antimicrob Agents Chemother.

[7] Mayxay M, Pukrittayakamee S, Newton PN, White NJ (2004). Mixed-species malaria infections in humans. Trends Parasitol.

[8] Imwong M, Nakeesathit S, Day NP, White NJ (2011). A review of mixed malaria species infections in anopheline mosquitoes. Malar J.

[9] Hawkins VN, Joshi H, Rungsihirunrat K, Na-Bangchang K, Sibley CH (2007). Antifolates can have a role in the treatment of Plasmodium vivax. Trends Parasitol.

[10] Khim N, Andrianaranjaka V, Popovici J, Kim S, Ratsimbasoa A, Benedet C (2014). Effects of mefloquine use on *Plasmodium vivax* multidrug resistance. Emerg Infect Dis.

[11] Ariey F, Witkowski B, Amaratunga C, Beghain J, Langlois AC, Khim N (2014). A molecular marker of artemisinin-resistant *Plasmodium falciparum* malaria. Nature.

[12] Ashley EA, Dhorda M, Fairhurst RM, Amaratunga C, Lim P, Suon S (2014). Spread of artemisinin resistance in *Plasmodium falciparum* malaria. N Engl J Med.

[13] Miotto O, Amato R, Ashley EA, MacInnis B, Almagro-Garcia J, Amaratunga C (2015). Genetic architecture of artemisinin-resistant *Plasmodium falciparum*. Nat Genet.

[14] Douglas NM, Anstey NM, Angus BJ, Nosten F, Price RN (2010). Artemisinin combination therapy for vivax malaria. Lancet Infect Dis.

[15] Gogtay N, Kannan S, Thatte UM, Olliaro PL, Sinclair D (2013). Artemisinin-based combination therapy for treating uncomplicated *Plasmodium vivax* malaria. Cochrane Database Syst Rev.

[16] Popovici J, Menard D (2015). Challenges in antimalarial drug treatment for vivax malaria control. Trends Mol Med.

[17] Popovici J, Kao S, Eal L, Bin S, Kim S, Menard D (2015). Reduced polymorphism in the Kelch propeller domain in *Plasmodium vivax* isolates from Cambodia. Antimicrob Agents Chemother.

[18] Yuan L, Wang Y, Parker DM, Gupta B, Yang Z, Liu H (2015). Therapeutic responses of *Plasmodium vivax* malaria to chloroquine and primaquine treatment in northeastern Myanmar. Antimicrob Agents Chemother.

[19] Malaria Consulting Committee (2002). Principles and regimens of antimalarial drug use in China. Chin J Parasit Dis Con.

[20] Li N, Parker DM, Yang Z, Fan Q, Zhou G, Ai G (2013). Risk factors associated with slide positivity among febrile patients in a conflict zone of north-eastern Myanmar along the China-Myanmar border. Malar J.

[21] Zhou G, Sun L, Xia R, Duan Y, Xu J, Yang H (2014). Clinical malaria along the China-Myanmar border, Yunnan Province, China, January 2011–August 2012. Emerg Infect Dis.

[22] Zhang Q, Lai S, Zheng C, Zhang H, Zhou S, Hu W (2014). The epidemiology of *Plasmodium vivax* and *Plasmodium falciparum* malaria in China, 2004–2012: from intensified control to elimination. Malar J.

[23] Zhou G, Lo E, Zhong D, Wang X, Wang Y, Malla S (2016). Impact of interventions on malaria in internally displaced persons along the China-Myanmar border: 2011–2014. Malar J.

[24] Bruce MC, Galinski MR, Barnwell JW, Snounou G, Day KP (1999). Polymorphism at the merozoite surface protein-3alpha locus of *Plasmodium vivax*: global and local diversity. Am J Trop Med Hyg.

[25] Yang Z, Miao J, Huang Y, Li X, Putaporntip C, Jongwutiwes S (2006). Genetic structures of geographically distinct *Plasmodium vivax* populations assessed by PCR/RFLP analysis of the merozoite surface protein 3beta gene. Acta Trop.

[26] Tamura K, Stecher G, Peterson D, Filipski A, Kumar S (2013). MEGA6: molecular evolutionary genetics analysis version 6.0. Mol Biol Evol.

[27] PlasmoDB: http://plasmodb.org/plasmo/. Accessed 1 Nov 2016.

[28] Hupalo DN, Luo Z, Melnikov A, Sutton PL, Rogov P, Escalante AV (2016). Population genomics studies identify signatures of global dispersal and drug resistance in *Plasmodium vivax*. Nat Genet.

[29] Nei M, Gojobori T (1986). Simple methods for estimating the numbers of synonymous and nonsynonymous nucleotide substitutions. Mol Biol Evol.

[30] Menard D, Khim N, Beghain J, Adegnika AA, Shafiul-Alam M, Amodu O (2016). A worldwide map of *Plasmodium falciparum* K13-propeller polymorphisms. N Engl J Med.

[31] Pearson RD, Amato R, Auburn S, Miotto O, Almagro-Garcia J, Amaratunga C (2016). Genomic analysis of local variation and recent evolution in *Plasmodium vivax*. Nat Genet.

[32] Putaporntip C, Kuamsab N, Kosuwin R, Tantiwattanasub W, Vejakama P, Sueblinvong T (2016). Natural selection of K13 mutants of *Plasmodium falciparum* in response to artemisinin combination therapies in Thailand. Clin Microbiol Infect.

[33] Zhang DD, Lo SC, Cross JV, Templeton DJ, Hannink M (2004). Keap1 is a redox-regulated substrate adaptor protein for a Cul3-dependent ubiquitin ligase complex. Mol Cell Biol.

[34] Mbengue A, Bhattacharjee S, Pandharkar T, Liu H, Estiu G, Stahelin RV (2015). A molecular mechanism of artemisinin resistance in *Plasmodium falciparum* malaria. Nature.

[35] Takala-Harrison S, Jacob CG, Arze C, Cummings MP, Silva JC, Dondorp AM (2015). Independent emergence of artemisinin resistance mutations among *Plasmodium falciparum* in Southeast Asia. J Infect Dis.

