# Supplementary material for: Genetic diversity of the Pvk12 gene in Plasmodium vivax from the China-Myanmar border area
Source: Malar J. 2016 Nov 4;15:528. doi: 10.1186/s12936-016-1592-z (PMC5096284; doi:10.1186/s12936-016-1592-z)
Supplement: Supplementary file 1 — Additional file 1. Linkage disequilibrium analysis of total samples. [file 12936_2016_1592_MOESM1_ESM.doc]

**Additional file 1.** **Linkage disequilibrium analysis of total samples.**

| Site1 | Site2 | Distance | D | D’ | R | χ^2^ |
| --- | --- | --- | --- | --- | --- | --- |
| 372 | 516 | 144 | 0 | -1 | -0.008 | 0.016 |
| 372 | 1080 | 708 | 0 | -1 | -0.008 | 0.016 |
| 372 | 2091 | 1719 | 0 | -1 | -0.012 | 0.039 |
| 516 | 1080 | 564 | 0 | -1 | -0.008 | 0.016 |
| 516 | 2091 | 1575 | 0 | -1 | -0.012 | 0.039 |
| 1080 | 2091 | 1011 | 0 | -1 | -0.012 | 0.039 |
