# Supplementary material for: Genetic diversity of the Pvk12 gene in Plasmodium vivax from the China-Myanmar border area
Source: Malar J. 2016 Nov 4;15:528. doi: 10.1186/s12936-016-1592-z (PMC5096284; doi:10.1186/s12936-016-1592-z)
Supplement: Supplementary file 2 — Additional file 2. Linkage disequilibrium analysis of the Tengchong samples. [file 12936_2016_1592_MOESM2_ESM.doc]

**Additional file 2. Linkage disequilibrium analysis of the Tengchong samples.**

| Site1 | Site2 | Distance | D | D’ | R | χ^2^ |
| --- | --- | --- | --- | --- | --- | --- |
| 372 | 516 | 144 | 0 | -1 | -0.02 | 0.042 |
| 372 | 1080 | 708 | 0 | -1 | -0.02 | 0.042 |
| 516 | 1080 | 564 | 0 | -1 | -0.02 | 0.042 |
